# Hospital and Institutional News

**Published:** 1913-09-13

**Authors:** 


### HOSPITAL AND INSTITUTIONAL NEWS.

### DUBLIN RIOTS: AN EYE-WITNESS' ACCOUNT OF THE INJURED IN HOSPITAL.

Just after we had gone to press last week the following account arrived from the Jervis Street Hospital, which we very much regret came too late for publication: "At eight o'clock on Saturday evening (August 30) the first of the injured arrived at Jervis Street Hospital, and soon the waiting-rooms of the dispensary were filled with men who had been batoned in the police charges. The majority had received scalp wounds. One civilian and one constable were admitted with broken legs, whilst of the others admitted one man died that night. On Sunday a fresh swarm arrived about 1.30 p.m., and continued to come all that evening and night. Saturday and Sunday were the worst days; in fact, the resident staff and all the available nurses were kept continually busy day and night on both days. Isolated cases have kept coming in since. Of those who have come back to be dressed only one has been septic so far. Well over three hundred persons were treated for injuries."

### YORK LABOUR AND THE COUNTY HOSPITAL.

The branch of the Independent Labour Party at York has passed a resolution to the effect that the findings of the recent Commission of Inquiry call for immediate action, and that, unless immediate steps are taken to carry out the suggestions of the Commissioner and to prevent the recurrence of such action, the party will strongly advise the working-men to abstain from contributing to the maintenance of the institution. We congratulate the York Labour men on the attitude of mind which led to the passing of this resolution, for such an attitude of intelligent interest in the standard of administration of the hospital in any community's midst is the only final guarantee of an adequate income and efficient work, as the infirmary and inhabitants of Worcester have found out for themselves during the past few weeks. The resolution passed last week by the Independent Labour Party at York is the swift and convincing proof of the contention which lay at the root of our article last week on "The Scarcity of Resident Medical Officers," namely, that "where it is difficult to obtain residents to-day it may be difficult to obtain patients to-morrow. For the unescapable truth remains that citizens in every locality deserve the hospital, good or bad, which they possess." Having been acquainted with this truth through many years of active hospital experience, we see in the York Labour Party's resolution not "the very foolish threat" which the frightened *Yorkshire Herald* shrinks from, but the first active signs of a quickening conscience on the part of the people of York. This sign has only to become general for York to have a hospital worthy of the name, for the past to be remembered only as a warning, and for all anxiety as to the hospital's income to be spared the hospital's administrators. The York Labour Party has set an example which, if followed up by other sections of the hospital's supporters, should produce a clean sweep of incompetence in six months.

### THE NEWCASTLE INFIRMARY CHAPEL.

We had to hold over from last week, owing to the extreme pressure on our space, a small but important item of news concerning the Royal Victoria Infirmary, Newcastle. At the quarterly meeting, held the previous week, the governors approved by a large majority the action of the house committee in re-opening the chapel. The history of the long controversy is now, we hope, fully ended. The house committee and governors should be free henceforth to devote their whole energies to the prime purpose of the institution, which is, it must never be forgotten, the cure of the sick through the adequate equipment and maintenance of the institution.

### SANATORIUM SCAREMONGERS IN DUBLIN.

It is the fashion at the present time to say that an intelligent Englishman always turns out on examination to be an Irishman, a German, or a Jew. Certainly exceptionally clever individuals of these races seem easier to name offhand than men of our own. Perhaps they advertise themselves better. But in any case the truth is that the greatest common measure of each nation shows the same number of wise men and fools. Look at the panic in Ireland about the infective dangers of consumption which is still making the United Kingdom laugh at such a shameless combination of cowardice and folly. A modern sanatorium for consumption, as we have often repeated, is not only no danger to a locality, it even tends to raise the rents. But the stupidity which cannot grasp the first of these facts is accompanied by a paralysis of the business faculties which cannot grasp the second, for folly is justified of her children on commercial as well as on other grounds. Thus last week the Dublin newspapers were reporting, under the suggestive title of "Another Peamount?" the terrible discovery that Lady Aberdeen had purchased a house and grounds seven miles from Dublin (a dreadful proximity, is it not?) with a view to establishing a large consumptive hospital, and the locality was urged to bestir itself to prevent her from doing so. Happily, the bubble collapsed after a free incision on the part of Sir Lambert Ormsby, senior surgeon to the National Children's Hospital, Dublin, and of Dr. E. J. McWeeney, Professor of Pathology and Bacteriology at University College, Dublin, who pointed out that the sanatorium is intended for cases of tubercular bones and joints requiring surgical treatment. Unable, of course, to grasp the difference between the pulmonary and non-pulmonary tuberculosis from the point of view of infection, the protesting Irishmen are feeling very much ashamed of themselves. Since, however, the hospital world cannot permit itself so cheap a victory over a few terrified laymen, we cannot forbear to point out that, the object of the sanatorium once known, "non-infectious" is an adjective likely to be used as recklessly as its opposite. The point to make, again, is not that one type of sanatorium is less "a danger" to the community than another, but that neither, under proper control, is a danger at all. Sea-sickness is not at present believed to be infectious. But why should we set limits to the beneficent discoveries of science? If it is considered infectious in the future, that will be no reason for preventing people from going on the sea. It will be a reason for providing proper isolation arrangements on every vessel, which is exactly what the sanatorium provides for infectious consumptives on land.

### "BORROWED PLUMES."

By a recent mail we have received the first number of a new monthly contemporary entitled *The Modern Hospital*, a journal devoted to the building, equipment, and administration of hospitals, sanatoriums, and allied institutions, and to their medical, surgical, and nursing services. Our new *confrère* presents an attractive appearance. Indeed, first numbers are usually attractive, but when we turned to the editorial staff and found thereon the names of Dr. Hurd, Dr. Frederic A. Washburn, Dr. Goldwater, Dr. Winford Smith, and Dr. Hornsby, one of the authors of a recently issued book bearing the same title as the new monthly, we understood how it had been possible to produce so practical and attractive a number. Dr. Hurd has won the confidence and affection of his contemporaries. Dr. Washburn has given evidence of great administrative power and invaluable practical knowledge, first as assistant and then as medical superintendent of the Massachusetts General Hospital, Boston. Dr. Goldwater is a prolific writer who has interested himself greatly in the planning of hospitals, and Dr. Hornsby's book shows a capacity for literary output on administrative questions which is rare. We wish *The Modern Hospital* every success and a long career of practical usefulness. We may, however, modestly observe, although our contemporary describes us as "so thoroughly conservative a journal," that there is one thing it cannot claim originality for, and that is its title, which we originated several years ago. Amongst its other interesting contributions this journal contains an article on the English National Insurance Act and its probable effects on the hospitals of the British Isles by Mr. Stewart Johnson, the well-known secretary of the Great Ormond Street Children's Hospital. The subscription is 14s. per annum, including postage, and the business office is Metropolitan Building, St. Louis, U.S.A.

### THE GROWTH OF SPECIAL DEPARTMENTS.

The London Hospital's Special Department, under Dr. James Mackenzie, is now at work for the treatment of heart cases. The pressure on this department has so increased that it has become necessary to appoint an assistant to Dr. Mackenzie, and Dr. John Parkinson has been appointed. A request has been received from the London County Council, asking the London Hospital to treat a certain number annually of dental cases, just as it now treats a certain number of aural, ophthalmic, and skin cases from the London County Council schools. The department will sit on five afternoons in the week, and special dental surgeons will be appointed.

### HOLIDAY SURGERY.

On account of the increasing number of surgical operations, the London Hospital has for the first time appointed two anæsthetists during the months of August and September, when the visiting anæsthetists are away for their holidays. Also, on account of the stress of work in the wards during the holiday season, the committee has appointed an extra emergency officer to assist wherever assistance is most required.

### THE FUTURE OF THE HUTCHINSON MUSEUMS.

We are hardly surprised that the wish, recorded in our columns recently, which was contained in Sir Jonathan Hutchinson's will that the museums founded by him should be disposed as he had desired during his lifetime, is immediately followed by the statement of a difficulty. The least complicated wishes in a testamentary document generally prove the most intricate to carry out, and in this case, we learn, no provision beyond that provided, and partly spent, during the donor's life exists to secure the museums' future. The Haslemere Museum is understood to be insured for £10,000, and the sum spent on the museums and their equipment is believed to be £30,000. Up to the present the annual cost of maintenance of the Haslemere Museum has been £400, and a guarantee fund is now asked for to provide this sum for the space of five years, during which period the form of its permanent maintenance can be decided on. It is pointed out that the important historical and geological galleries at Haslemere are arranged on the "space-for-time" system, and that though this would doubtless be preserved were it found necessary ultimately to hand over the museum to some public body—the Surrey Education Committee, for example—yet the habitat of the museum, under such a transference of control, would almost surely not be Haslemere, wherever else it might be found. Surely Haslemere sufficiently understands the value of the great man who made it famous not to allow his most cherished local achievement to pass out of its hands for the mere want of £400 a year, or a capital sum of only £10,000?

### LATE INSPECTOR-GENERAL OF HASLAR HOSPITAL.

It is only eight months since the honour of K.C.B. was conferred on Sir Duncan Hilston, who has now died, aged seventy-six. Well known to the Navy Medical Service as Inspector-General of Hospitals and Fleets from 1892-1897, for three years of this period he was in active charge of Haslar Hospital. A St. Andrews man, he graduated M.D. at that University in 1858, and saw active service in New Zealand and in Abyssinia, for which he received the medal in each case. In 1902 the Civil C.B. was bestowed upon him, and two years later he was appointed as honorary physician to King Edward.

### AN INFIRMARY SUPERINTENDENT AND THE LIMITED PANEL.

The treatment of the residential staffs during sickness at the various institutions of the Southwark Board of Guardians has exemplified the annoyances caused by the Insurance Act. At the infirmary, Dr. Bruce, the medical superintendent, has attended the staff, but, much against his will, he has been compelled to go on the panel in order that the present arrangements can be continued. Dr. Bruce, in a letter to the Board, informed the Guardians that the London Insurance Committee had insisted on the insured staff selecting their doctor, and he had, therefore, been compelled to notify his willingness to attend the staff, as he had done in the past. The Guardians, in approving Dr. Bruce's action, decided that the dispensers should not be allowed to go on the chemists' panel. In future the insured resident staffs at the various institutions will have to obtain their medicines, etc., from outside chemists who are on the panel, instead of from the Guardians' dispensaries. With regard to the three workhouses and their staffs, the medical officers of Christchurch and St. George's Workhouses have gone on the panel, but the medical officer at Newington Workhouse, where there is a very large staff, has not. As these are only part-time officers, they are at liberty to choose what course they shall take, but the difficulties of administration will be increased at Newington Workhouse if two or three outside doctors are called in to attend members of the staff during illness.

### A PLAGUE OF FLIES IN A LONDON INFIRMARY.

The serious outbreak of enteritis at Southwark Infirmary, apparently due to the proximity of dust heaps under the control of the Camberwell Borough Council, referred to in The Hospital on August 30, has been followed by the deaths of three children. There have been twenty-eight cases, and, to add to the torture of the patients, the institution has been infested with a plague of flies. They have not only invaded the kitchen and store rooms, and, consequently, had the run of the food, but also the wards. The patients have to wear gauze veils to protect their faces, and although a fortnight has elapsed since Dr. Fletcher, a medical officer at the Local Government Board, visited the infirmary, no report, as we write, has yet been presented. An inspector has visited the dust heaps, and described them publicly as "objectionable." He is now, we understand, conferring with the Camberwell Sanitary Authority. The Borough Council has placed a tarpaulin screen on one side of the trucks, which in certain winds becomes a guiding sheet for the dust to the institution windows. At their meeting last week the Guardians were indignant at the delay of the Local Government Board in dealing with such a serious matter, and they decided that the clerk should send a letter by special messenger to Whitehall to see if something could not be done to speed matters up. Should the delay continue, the Guardians will have to tackle the matter themselves, for when a public nuisance develops into a public danger wise men refuse to brook official delay. If this would mean a technical legal offence public opinion can be relied on to demand an act of indemnity afterwards. The next official event will be the meeting of the Works and General Purposes Committee of the Camberwell Borough Council on September 15. The Council is not expected to meet before September 24.

### SYPHILIS NOTIFICATION ON A RECIDIVIST SYSTEM.

When a lay paper, even if a relatively expensive monthly, attempts to deal with a question so technical from several points of view as the control of syphilis it is almost certain to go wrong. The article in the current *English Review*, which reads as if a medical author had been asked to "write down" to the laity, is hardly an exception. The right way to deal with a tabooed subject is not to emphasise how "difficult" it is. Such attempts to disarm criticism only make readers feel that they are expected to be shocked. Adjectives of either variety should be scrupulously avoided, and the subject discussed as if neither praise nor blame was fashionable concerning it. Apart from this cardinal fault in style the article makes a definite suggestion which is our excuse for referring to it here. "There should be," says the writer, "a system of medical notification somewhat on the lines of the recidivist police system." The advantages of this plan are described as "absolute professional secrecy," while the threat of exposing defaulters who failed to report themselves is put forward as probably sufficient to prevent evasion. Perhaps it may be. Unfortunately, however, not one out of a hundred readers of *The English Review* has an adequate notion of how the recidivist police system is administered, and as that is the case with the readers of a shilling magazine how much chance is there of gripping the large mass of readers of halfpenny newspapers, which will probably quote the article for the sake of its sensational element? These are the people, however, who have to be got at, and we respectfully suggest that if Mr. Austin Harrison is serious in his wish to do an important editorial service to the country and high-class journalism, he should induce his author to try again and to develop the one small pinch of salt which otherwise almost certainly will be buried at the foot of his contributor's article.

### A HOSPITAL EX-OFFICIAL SENTENCED.

From an institutional point of view, the chief interest attaching to the trial last week of the two Wagners, mother and son, at the Central Criminal Court for blackmail resides in the fact that the latter was at one time employed as a clerk at St. George's Hospital, London. From that institution he absconded with £15 12s. belonging to certain patients, and with a watch belonging to a police constable who was undergoing treatment in the hospital. Eventually, it seems, the mother went to the hospital and made a partial restitution. She, however, is stated by the police to be the active partner in the life of crime which the Wagners carried on successfully and profitably; and the jury took the same view by recommending the young man to mercy. He accordingly received a less severe sentence than his mother did. Robberies in hospitals are not by any means rare; but are nearly always the work of outside operators, who find in the crowds of visiting days or the excuse of an injured relative convenient means of obtaining access to portable property belonging to other people. But it is exceedingly uncommon for dishonesty to be perpetrated by any of the officials of a hospital.

### LEGACIES, SISTERS, AND MEDICAL MEN.

Testamentary bequests to nurses, other than such as may have nursed some patient through a long illness "in private," are very infrequent events. Not long ago we had occasion to note Mr. Fry's legacies to the hospital staff at Bristol (The Hospital, August 9, 1913, p. 550). Now it is the turn of a nursing-home sister to receive similar recognition. By the will of the late Duke of Sutherland an annuity of £250 is payable "to Lily Watson, head nurse at the nursing home, 15 Henrietta Street, W., in recognition of her kindness, skill, and care after a serious operation I underwent." We congratulate the lady concerned as much upon the outspoken compliment thus conveyed as upon the receipt of this handsome bequest. As a minor matter of interest, we note also that no surgeon or doctor receives from the Duke any such testimonial or legacy, although the deceased nobleman's valet gets two pounds a week for life.

### THE MEDICAL DEFENCE UNION AGAIN.

The latest case which is being brought by this admirable body, though still before the courts, is worth attention. A picture framer and a servant girl, aged twenty, have been charged at Lambeth Police Court with blackmailing Dr. William Jarvis Bell. It is alleged that Dr. Bell has been accused of immoral relations with the female defendant, to whom the male defendant is stated to be engaged to be married. The prosecution was on behalf of the Medical Defence Union. In the witness-box the male defendant affirmed that on August 13 the servant told him that she had been insulted by the doctor, to whom she had written a letter in the witness's name threatening to put the matter in the hands of a solicitor. On being taken to a police station she made a complaint to an inspector. In cross-examination the witness alleged that he had never seen the threatening letter. He admitted that he was hard up and in want of employment, but denied that he knew the servant had asked Dr. Bell for £100. The defendants have been committed for trial, so pending the result of the case we refrain from comment.

### MILITARY HOSPITALS AND VOLUNTARY AID.

The Royal Army Medical Corps Territorial Force (Aberdeen) have now completed again a year's training. The camps seem to have proved very serviceable for the training of the voluntary service in the care of the sick and wounded. The 1st Scottish General Hospital R.A.M.C. (T.) camped at Woolwich, during which time they received training in the Royal Herbert Hospital. Before going into the hospital it was clearly understood that each man was to learn the most minute details of the work set to him. Major Ensor inspected each man daily, asking questions on the work of each day. At the end of the period an inspection was made by Major-General Whitehead, who, we understand, seemed very pleased at the amount of work learned by the unit. The 1st and 2nd Highland Field Ambulance R.A.M.C. (T.) underwent the training at Denny, Stirlingshire. Their inspection was made by Major-General O'Keefe, who also congratulated the Lieutenant-Colonel on the condition of his men.

### THE ATHLETIC SPORTS BOGEY AGAIN.

Mr. Frederic Harrison's vigorous letter to the *Times* protesting against the collecting of large sums of money to train British representatives in the Olympic Games at Berlin will appeal to many medical men who remember with enjoyment their athletic career at hospital, but who would have disliked turning themselves, even temporarily, into professional running machines. We remember, some dozen years back, seeing Dr. H. A. Munro competing in a long distance event at the English Championships at Stamford Bridge. Rather pale and worn looking, and wearing glasses, he gave by his appearance the impression of having snatched a little time from his duties at Guy's Hospital for diversion of himself and others. As it happened he won in very good time. Nowadays a competitor like this would probably be just beaten by some half-crazed American in the hands of a low type of trainer. England, as the Germans with characteristic veracity assure us, is the motherland of sport. We might well be satisfied with the distinction and with the compliment other nations have paid us in following our lead, and decline to imitate in turn a transatlantic people with more money and resources than real wisdom, which childishly makes a business of pleasure and, also childishly, fails to see the necessity of playing fair. These are hard words, but the praiseworthy letters of some Americans of the better class written to English newspapers at the time of the last Olympic Games quite bear them out. In a note on the "Lessons of the Fifth Olympiad" which appeared in these columns on August 10, 1912, we summed up the whole question and prescribed the remedy.

### THE HISTORY OF RUBBER OPERATING GLOVES.

Nowadays rubber operating gloves form no inconsiderable item in the expenses of a hospital operating theatre: in the last ten years their use has increased in this country from the exceptional to the almost invariable. The history of their evolution has lately been given to the world by Professor Halsted, of Baltimore, the renowned American surgeon, who took a foremost share in evolving the modern operation for cancer of the breast. In the winter of 1889-90 Professor Halsted's theatre sister suffered from a dermatitis set up by the prolonged immersion of her hands and arms in solutions of mercuric chloride. He was very unwilling to be deprived of her services, and the idea struck him that thin rubber gloves might serve to protect her skin. The experimental gloves made to his order were so successful that soon the assistants began to wear them too for the same reason. Then Professor Halsted began to wear them himself whenever he was to open a joint. Later on as his assistants became operators in their turn they found the habit of wearing gloves had got so ingrained that they lost no manipulative dexterity by it, and some even felt at a loss without the gloves. Dr. Bloodgood was the first of these pupils of Professor Halsted's to make an invariable practice of wearing gloves in the interests of antisepsis: this was in December 1896.

### MENTAL HOSPITALS AND PELLAGRA.

When the first few cases of pellagra detected in this kingdom were recorded a few months ago they were mostly from Scottish mental hospitals, and there was a tendency to scoff at the confident predictions of Dr. Sambon and others that the disease would be found widely spread in Britain. It is every day, however, becoming more obvious that this prediction was accurate, and that pellagra is by no means so extremely rare as it was supposed even a year ago to be. Thus in the *Lancet* of September 6 Dr. Blandy, of Napsbury Mental Hospital (Middlesex), publishes no fewer than eleven cases from that institution, all verified as clear cases of indubitable pellagra. This and the similar discoveries elsewhere in Britain should finally dispose of the old theory that pellagra is due to eating infected maize; for that cereal is almost unknown in Britain as an ingredient of diet for human beings. This, of course, does not prove that Dr. Sambon's theory of infection conveyed by the bite of a sandfly (*simulium*) is correct; but it is interesting that a search in the banks of the River Colne, which flows within seven hundred yards of Napsbury institution, resulted in the discovery of the larva of this fly, although adult specimens were not found. Dr. Blandy's patients all exhibited severe symptoms in or about the month of July; and he asks whether this can be coincidence, or the result of some more definite factor.

### MADAME SARAH BERNHARDT AND OUR HOSPITALS.

An interesting ceremony took place on Saturday last at the Carlton Hotel, when Madame Sarah Bernhardt was presented with bouquets in token of her sympathy with and work for the Charing Cross Hospital and the French Hospital. Readers of The Hospital will recall that Madame Bernhardt visited both these institutions when last in London, and that she is taking an active interest in the benefit performance at the Coliseum on October 11 next. The King and Queen are expected to be present, and the occasion will be the first on which their Majesties have honoured a dramatic performance for the benefit of hospitals with their presence. In view of the keen interest which Madame Bernhardt has taken in the event, and in gratitude for her generosity in giving her very valuable services, Mr. A. C. Fotheringham Lysons, secretary of the special appeal for Charing Cross Hospital, presented Madame Bernhardt with a bouquet of carnations. A similar presentation was made on behalf of the French Hospital by Dr. Louis Vintras, a member of the medical staff. Madame Bernhardt, in acknowledging these gifts, expressed her pleasure at being able to help. At the performance on October 11, in addition to Madame Sarah Bernhardt, the cast will include Miss Ellen Terry, who will return to the stage for this special occasion, and who, by the way, received a great ovation on taking her seat in the stalls at the first performance of "Androcles and the Lion" last week. Boxes are sold at 100 guineas each, and the cheapest seats are 5s., and are nearly all sold.

### A MEDICAL SCIENTIST'S DEATH AT SEA.

Dr. Tempest Anderson, of York, the ophthalmic surgeon, and formerly Tindall Lecturer on Volcanoes at the Royal Institution, has died from enteric fever on his way home from the Philippines. Born in 1846 and educated at York, University College, London, and London University, Dr. Anderson, whose father, by the way, was also a medical man, graduated not merely M.D., but also B.Sc. He early showed a preference for seismography, and the earthquakes in the West Indies during 1902 and 1907 led to him compiling a joint report. His appointments and positions of note include those of consulting ophthalmic surgeon to the York County Hospital, vice-president of the British Association, president of the Museums Association and of the Yorkshire Philosophical Society. Indeed the York Museum owes to his advocacy a new lecture hall, while he was a member of the Council of the Geological and Royal Geographical Societies. He was also Sheriff of York in 1894, and his wide interests included the preservation of open spaces and the housing problem, while his recreations were almost only of a scientific kind. As a member of the Alpine Club he enjoyed mountaineering, not merely as a sport, but for the opportunities which it gave him for photographing volcanoes, craters, and mountain phenomena generally; he was also a mechanician, especially in relation to optics, and a cyclist. The papers of his on ophthalmology are known to the interested student, and in 1902 he published "Volcanic Studies." He was unmarried, and a striking instance of that still not too common combination—man of science and medical practitioner.

### THE PROGRESS OF CREMATION.

It is interesting to note the very slow but still steady increase in the number of cremations. During the past year there were 1,064, which is the largest number recorded since 1885, when cremation was first practised in the United Kingdom. Golder's Green records 521, Manchester 149, Liverpool 52, Glasgow 44, Birmingham 40, Woking 15, and so on. The kind of fact, however, which is most likely to influence the public is the wish expressed by the late Duke of Sutherland, in the recently published will, to be cremated. The will, also, was noteworthy for the statement that he desired his funeral to be "conducted in as simple a manner as possible," a desire only too rare among all classes to-day. Perhaps it should be added that directions for cremation, and for a simple funeral for the matter of that, are better expressed in a letter to the executors than in a will, which is often not opened till the funeral is over.

### ATTRACTIONS OF INFIRMARY RESIDENT POSTS.

To meet the needs of readers who may wish for further information as to the worth of resident appointments in Poor-Law infirmaries than Dr. Thackray Parson's article last week had space to give, we may add that in some infirmaries the junior medical officer is appointed for six months only, but the appointment is always extended without any difficulty if his work has been carried out satisfactorily. In a few infirmaries the scale of salaries is higher. At Camberwell Infirmary, for instance, the Board of Guardians has recently adopted the following scale subject to the approval of the Local Government Board. The senior assistant medical officer is to be paid £230, rising to £260; the second £200, rising to £215; the third £180, rising to £190; the fourth £160, rising to £170; and the junior £140. The Wandsworth Union has also recently adopted a new scale of remuneration for its assistant medical officers. Another advantage of the Poor-Law infirmary resident's work is that when he is not giving the anæsthetic the medical officer will usually act as the operator's assistant, and he thus sees the details of the operation in a way in which the medical student gets few opportunities. All the minor operations are usually performed by the medical officers themselves, and if keen upon operative surgery the medical superintendent is usually willing to let them perform some of the major operations under his supervision if he is satisfied that they can do so efficiently. Again, as regards mental experience the infirmary medical officer sees many cases, including delirium tremens, puerperal insanity, and general paralysis of the insane; and, although he is not able to follow many of them to their conclusion, he is able to gain a practical knowledge of diagnosis and of the legal requirements and difficulties of certification. The experience to be gained in these wards is just that which is most useful to the man in general practice.

### SALARIES FOR VACCINATION OFFICERS.

The Local Government Board has sanctioned the payment of salaries in lieu of fees to their vaccination officers. Through the great increase in the number of conscientious objectors to vaccination, the fees of the officers, of course, has fallen off very considerably, and at first it was suggested that they should receive fees for recording those obtaining exemption certificates. By this means, which had rather a Gilbertian air about it, their remuneration would have been something approaching what it was in 1908, when the Vaccination Act came into force. The Local Government Board, however, declined to entertain such a proposal, and the Guardians would only resort to permanent salaries. These have been based on the average fees received by the officers during the five years ending December 31, 1907. The Board has always objected to this method of payment heretofore, as it tended to remove the inducement for the officer to secure the highest possible number of vaccinations. The salaries are to be revised in the event of the combined number of vaccinations and exemptions in a district falling below a minimum of 80 per cent. of the number of births registered.

### THE PHARMACIST'S ADAPTABILITY.

We have frequently had pleasure in emphasising the adaptability of institution pharmacists, an adaptability undoubtedly due to the character of their professional training. A brilliant example recently presents itself in the person of Mr. H. C. T. Gardner, M.P.S., F.C.S., who has resigned his appointment at the Miller Hospital, Greenwich, after a distinguished career in London, to take over the management of the pharmaceutical department of a large firm in Calcutta. Mr. Gardner, who qualified in 1899, was chairman of the Public Pharmacists' Association for three years (1906-7-8), and is regarded as an authority on *x*-rays, radium, and rubber. He is also a prolific writer on these subjects. His colleagues will watch his future with interest, for this is only the latest example of the enlarging influence of institution life.

### THIS WEEK'S DRUG MARKET.

Business in the Drug market continues very dull and few price changes of interest have taken place. The position of opium is practically unchanged, but the impression prevails that prices are likely to decline. Makers of morphine have reduced their quotations, as also have the makers of codeine. The price of citric acid is still tending upwards. There has been as yet no further advance in the price of quinine. Iodine and iodides are firm at the late advance. At the recent public sale of drugs in Mincing Lane higher prices were paid for senna and cardamoms.

### TO OUR READERS.

Contributions are specially invited from any of our readers to these columns. They should deal with topical subjects and news. They must be authenticated for the information of the Editor only. The minimum payment if published is 5s. There is no hard-and-fast rule as to space, but notes of about twenty lines in length are preferred.

